# Giant Diverticulum of the Duodenum

**DOI:** 10.4021/gr359w

**Published:** 2011-11-20

**Authors:** Matthijs ter Horst, Marieke C. Hovinga-de Boer, Menno H. Raber, Joost M. Klaase

**Affiliations:** a Department of General Surgery, Medisch Spectrum Twente, Enschede, the Netherlands; b Department of Radiology, Medisch Spectrum Twente, Enschede, the Netherlands

**Keywords:** Small bowel diverticulum, Duodenum, Giant diverticulum, Gastrointestinal diverticula

## Abstract

A 50-year old female presented herself with abdominal bloating and pain in the Emergengy Department. The symptoms persisted and a clinical evaluation was made. A lesion suspect for a giant duodenal diverticulum was seen on the CT-scan, which was confirmed by enteroclysis. Surgical resection was performed. The diagnosis was histological confirmed after surgery. Small bowel diverticula are relatively common, with an estimated 5 - 22% incidence in the healthy population. They are usually asymptomatic, but can present with abdominal pain and weight loss. Complications such as bleeding and perforation can occur. Surgical resection is the treatment of choice in symptomatic patients.

## Introduction

Diverticula can be present anywhere in the gastrointestinal tract and can be either congenital or acquired. It’s a well known clinical entity in the colon, but relatively rare in the small intestine. When present in the small intestine the most common place for diverticula is the duodenum [1-5]. Diverticula occur at weak spots of the duodenal wall, such as the entry site of the common bile duct, the pancreatic duct and perivascular connective tissue sheath [2-6]. Duodenal diverticula are usually asymptomatic. When symptomatic, the most common and often only symptom is persisting abdominal pain. Duodenal diverticula need clinical attention because they carry the risk of serious complications. Complications such as gastrointestinal bleeding, biliary or pancreatic duct obstruction, obstructive ileus and perforation have been reported [1-7]. Diagnosis can be facilitated by making upper gastrointestinal radiographic studies in combination with endoscopic studies [2-6]. We present a 50-year-old woman with a giant diverticulum (6.8 x 4.5 x 1.8 cm) of the duodenum presenting herself with abdominal bloating and pain. The diagnosis was histologically confirmed after surgery. The relevant literature is reviewed.

## Case Report

**Figure 1:**
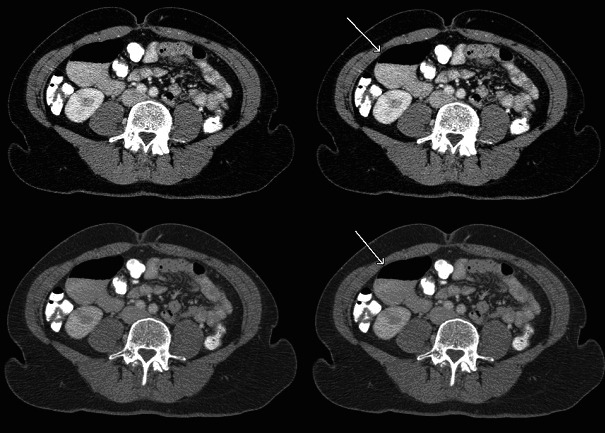
Distension of the duodenum with an air-fluid level.

A 50-year-old woman presented herself with abdominal bloating and pain in the Emergency Department. Her complaints had been present since several years and were thought to have started after a laparoscopic left nefrectomy, which had been done to facilitate a life-donor kidney donation. Clinical evaluation with laboratory analysis, conventional abdominal radiography and ultrasonography did not yield a clear cause for her complaints. After administration of oral and intravenous iodinated contrast material, a Computed Tomography (CT) scan of the abdomen was performed. It showed a distension of the pars descendens of the duodenum, 6.8 by 4.5 centimeters with an air-fluid level. The wall showed no abnormalities: no enhancement or loco regional fatty infiltration. Signs of food impaction or obstruction could not be observed (Fig. 1).

**Figure 2:**
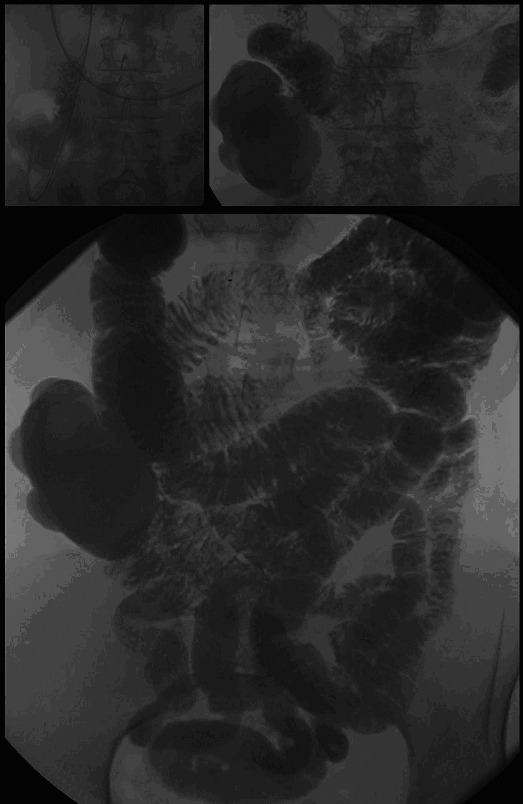
Giant diverticulum originating from the duodenum.

Six months later, a small bowel double contrast enteroclysis with barium suspension was performed. Placement of the gastroduodenal catheter into the duodenum pars horizontalis failed: it ended in a large diverticulum of the pars descendens of the duodenum, which was situated in the right lower quadrant of the abdomen. The double contrast series confirmed the diagnosis giant duodenal diverticulum (Fig. 2). This examination showed a normal aspect of the jejunum, and ileum. No signs of intraluminal pathology of the small bowel or obstruction were observed. Patient was discussed in the multidisciplinary gastrointestinal committee and a laparoscopic resection of this diverticulum was decided on.

**Figure 3:**
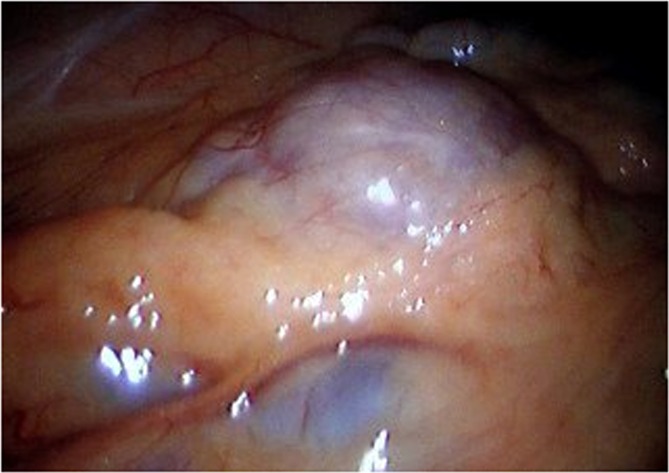
During laparoscopy a large protrusion of the duodenum was seen, projecting underneath the mesocolon.

**Figure 4:**
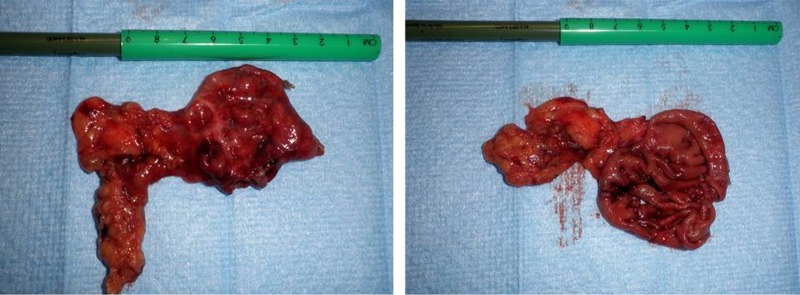
Resected diverticulum, left intact. Right the diverticulum was opened, showing normal duodenal mucosa.

Patient underwent a laparoscopy during which a large protrusion of the duodenum was seen, projecting underneath the mesocolon (Fig. 3). A window was made through this mesocolon after which the diverticulum was dissected using ultracision. The diverticulum was then resected by stapling it off at the neck (Fig. 4). Microscopic evaluation of the resected diverticulum showed a diverticulum of the duodenum without any signs of dysplasia or malignancy. Post operative the patient recovered without any complications.

## Discussion

Small bowel diverticula can be either congenital or acquired, with Meckel’s diverticulum being the only congenital form. Meckel’s diverticulum is a true diverticulum, mostly located on the antimesenteric border of the small bowel within 40 - 80 cm of the ileocecal valve. All other small bowel diverticula are acquired or false diverticula. False diverticula are bulging pouchlike herniations of the intestinal wall consisting of mucosa, submucosa and serosa, without a tunica muscularis [4]. Duodenal diverticula are the most common small bowel diverticula, with an estimated incidence of 5 - 22% in the healthy population [6]. The most common sites for them to occur are weak spots in the intestinal wall, such as the entry site of the common bile duct, the pancreatic duct and perivascular connective tissue sheath [2-6]. If a diverticulum arises within a radius of 2 - 3 cm from the ampulla of Vater they are called juxtapapillary diverticula [8-10]. A duodenal diverticulum is usually asymptomatic, but when symptoms occur the most common, and often only symptom is persisting abdominal pain. This a specific symptom makes it difficult for clinicians to diagnose a duodenal diverticulum. The importance of diagnosing a symptomatic duodenal diverticulum is because they carry the risk of complications, such as bleeding, obstruction of the biliary or pancreatic duct, obstructive ileus and perforation [1-7, 11].

Diagnosis of primary small intestinal disease is difficult and ultrasonography, CT, magnetic resonance imaging (MRI), capsule endoscopy and traditional endoscopy are all used. A double contrast enteroclysis is an important and valuable diagnostic procedure for evaluating structural abnormalities of the small bowel. The advantages are simplicity, availability, high diagnosis accuracy and low price [5, 12-14].

Many diagnostic examinations such as enteroclysis, CT, scintigraphy and a video capsule are helpful to verify the existence, location, size and amount of diverticula [5, 15]. After the 1970s, the widespread use of endoscopic retrograde cholangiopancreatography (ERCP) led to increased diagnosis of juxtapapillary duodenal diverticula [16]. Endoscopic techniques have been described as having replaced enteroclysis and to be the gold standard in diagnosing duodenal diverticula [15, 17]. CT has been reported as having a 71% sensitivity as compared to barium contrast studies [7]. De Vries et al. report a sensitivity of 36% and a specificity of 100% of CT in the diagnosis of juxtapapillary duodenal diverticulum [15].

However, a superiority of any modality to diagnose duodenal diverticular disease has not been described unambiguously. As in our case, diagnosis can be performed easily at CT [8]. On MRI, juxtapapillary diverticula can also be visualized on all consecutive imaging sequences. Juxtapapillary diverticula show both on CT and MRI pouches of the duodenal wall [9]. The most common finding is an air-fluid level in the diverticulum [8-10]. On CT, the fluid component consists of hyperdense oral contrast. On T2-weighted MRI images, the air-fluid level can be observed as a hyperintense fluid level with signal void level above [9]. Nevertheless, air is not always visualized within the diverticulum. A fluid-filled diverticulum has a well-defined rounded appearance. Confidence is especially high when continuity with the lumen of the duodenum is established by the presence of contrast material or when a connection with the duodenum is seen [8]. In our patient, an air fluid-level could be observed in the diverticulum which also showed connection with the duodenum. In enteroclysis, a contrast agent is instilled into the small bowel via a gastroduodenal catheter which is placed just beyond the ligamentum of Treitz. In this way, greater luminal distention and better depiction of the individual small bowel loops is reached [13].

Small bowel diverticula can be confused with Meckel diverticula. Meckel diverticula have been demonstrated by enteroclysis reflecting the morphology of the anomaly. A Meckel diverticulum is a blind sac which is attached to the antimesenteric border of the distal small bowel not connected with the umbilicus. Acquired diverticula lie on the mesenteric border [18].

The use of MR enteroclysis should be restricted to follow-up examinations of patients with well known small-bowel disease [14]. A critically appraised topic (CAT) compared MR enteroclysis (MRE) with CT enteroclysis and the gold standard of conventional enteroclysis (CE) for diagnosing small bowel Crohn’s disease and small bowel neoplasia. In overall diagnostic yield, MRE performed better and added extraluminal detail. CE performed better in detection of subtle mucosal detail [19]. Diverticula should always be noted and their significance must be evaluated together with other radiologic findings and the clinical status. Especially because it can simulate pancreatic pathology, recognition and differentiation of duodenal diverticula is very important [8].

There is only a need to treat a duodenal diverticulum when it becomes symptomatic or causes complications [1-7, 11, 20, 21]. Asymptomatic diverticula are innocent, and there are no reports about duodenal diverticula converting into neoplasms. Therefore it is permitted to leave intraoperatively found duodenal diverticula in situ when the symptoms of the patient cannot be explained by the diverticulum [20]. The treatment of choice for symptomatic diverticula mainly depends on the severity of the symptoms. Because of the rare appearance of symptomatic duodenal diverticula, there are no detailed recommendations or guidelines about the treatment of them. Surgical resection is the most common approach, but there have been a few reports of successful conservative management with antibiotics and percutaneous drainage [6, 20].

Concerning surgical resection, there is a wide range of surgical options available to treat a duodenal diverticulum. Reports have been made about local resection, but conversion to a Billroth II reconstruction or pylorus preserving pancreaticoduodenectomy has been described in case of a substantial inflamed duodenum [6, 21]. Most frequently, resection of the diverticulum after Kocher manoeuvre with one- or two-layer closure of the duodenum has been described [6, 21]. The open procedure has been described the most, but can also be performed laparoscopic depending on the surgeon’s preference [22]. It can be useful to place drainage tubes after surgery, especially when the retroperitoneum is affected by inflammation. Furthermore, a omentoplasty can be patched over the closure site [6]. During the procedure special care should be taken to avoid injury to the pancreatic duct and parenchyma as well as to the extrahepatic bile ducts, because most diverticula arise in the periampullary region [3, 21]. Injury to these structures can be avoided by placing a tube into Vater’s papilla before dissecting the diverticulum [6].

### Conclusion

Small bowel diverticula are a relatively common pathological entity in the healthy population. They are usually asymptomatic, but can present with persisting abdominal pain. They require attention of a clinician because complications such as mechanical obstruction and perforation can occur. Surgical resection of a symptomatic duodenal diverticulum is the definite treatment.

## References

[1] Chiu EJ, Shyr YM, Su CH, Wu CW, Lui WY (2000). Diverticular disease of the small bowel. Hepatogastroenterology.

[2] Chugay P, Choi J, Dong XD (2010). Jejunal diverticular disease complicated by enteroliths: Report of two different presentations. World J Gastrointest Surg.

[3] Sakurai Y, Miura H, Matsubara T, Imazu H, Hasegawa S, Ochiai M (2004). Perforated duodenal diverticulum successfully diagnosed preoperatively with abdominal CT scan associated with upper gastrointestinal series. J Gastroenterol.

[4] Kouraklis G, Glinavou A, Mantas D, Kouskos E, Karatzas G (2002). Clinical implications of small bowel diverticula. Isr Med Assoc J.

[5] Mantas D, Kykalos S, Patsouras D, Kouraklis G (2011). Small intestine diverticula: Is there anything new?. World J Gastrointest Surg.

[6] Martinez-Cecilia D, Arjona-Sanchez A, Gomez-Alvarez M, Torres-Tordera E, Luque-Molina A, Valenti-Azcarate V, Briceno-Delgado J (2008). Conservative management of perforated duodenal diverticulum: a case report and review of the literature. World J Gastroenterol.

[7] Yokomuro S, Uchida E, Arima Y, Mizuguchi Y, Shimizu T, Kawahigashi Y, Kawamoto M (2004). Simple closure of a perforated duodenal diverticulum: "a case report". J Nihon Med Sch.

[8] Stone EE, Brant WE, Smith GB (1989). Computed tomography of duodenal diverticula. J Comput Assist Tomogr.

[9] Jayaraman MV, Mayo-Smith WW, Movson JS, Dupuy DE, Wallach MT (2001). CT of the duodenum: an overlooked segment gets its due. Radiographics.

[10] Balci NC, Akinci A, Akun E, Klor HU (2003). Juxtapapillary diverticulum: findings on CT and MRI. Clin Imaging.

[11] Peixoto P, Amaro P, Sadio A, Figueiredo P, Almeida N, Gouveia H, Coutinho L (2010). A strange duodenal lesion. Rev Esp Enferm Dig.

[12] Zhan J, Xia ZS, Zhong YQ, Zhang SN, Wang LY, Shu H, Zhu ZH (2004). Clinical analysis of primary small intestinal disease: A report of 309 cases. World J Gastroenterol.

[13] Levine MS, Rubesin SE, Laufer I (2008). Pattern approach for diseases of mesenteric small bowel on barium studies. Radiology.

[14] Silit E, Basekim CC, Mutlu H, Kizilkaya E, Yigitler C (2011). Diagnosis of small-bowel disease: comparison of magnetic resonance enteroclysis and conventional enteroclysis. J Int Med Res.

[15] de Vries JH, Duijm LE, Dekker W, Guit GL, Ferwerda J, Scholten ET (1997). CT before and after ERCP: detection of pancreatic pseudotumor, asymptomatic retroperitoneal perforation, and duodenal diverticulum. Gastrointest Endosc.

[16] Chen Q, Li Z, Li S, Ding X, Liu Z, Wu C, Gong J (2010). Diagnosis and treatment of juxta-ampullary duodenal diverticulum. Clin Invest Med.

[17] Bach AG, Lubbert C, Behrmann C, Surov A (2011). Small bowel diverticula - diagnosis and complications. Dtsch Med Wochenschr.

[18] Maglinte DD, Elmore MF, Isenberg M, Dolan PA (1980). Meckel diverticulum: radiologic demonstration by enteroclysis. AJR Am J Roentgenol.

[19] Ryan ER, Heaslip IS (2008). Magnetic resonance enteroclysis compared with conventional enteroclysis and computed tomography enteroclysis: a critically appraised topic. Abdom Imaging.

[20] de Lange DW, Cluysenaer OJ, Verberne GH, van de Wiel A (2000). [Diverticulosis of the small bowel]. Ned Tijdschr Geneeskd.

[21] Schnueriger B, Vorburger SA, Banz VM, Schoepfer AM, Candinas D (2008). Diagnosis and management of the symptomatic duodenal diverticulum: a case series and a short review of the literature. J Gastrointest Surg.

[22] Yoneyama F, Miyata K, Ohta H, Takeuchi E, Yamada T, Kobayashi Y (2004). Excision of a juxtapapillary duodenal diverticulum causing biliary obstruction: report of three cases. J Hepatobiliary Pancreat Surg.

